# Development and validation of a multimodality radiomics-based nomogram for predicting HER2 expression status in invasive breast cancer

**DOI:** 10.3389/fonc.2026.1829333

**Published:** 2026-07-14

**Authors:** Xianwei Yang, Xiaoling Liu, Shenghong Wan, Yilin Luo, Mengjun Shen, Xin Liao

**Affiliations:** 1 Department of Radiology, Affiliated Hospital of Guizhou Medical University, Guiyang, China; 2 The People’s Hospital of Qixingguan, Bijie, China; 3 Dushan county People’s Hospital, Qiannan Buyi and Miao Autonomous Prefecture, Duyun, China; 4 Department of Radiology, Guizhou Hospital of The First Affiliated Hospital of Sun Yat-sen University (FAH-SYSU), Guiyang, China

**Keywords:** HER2 expression, invasive breast cancer, multimodality, nomogram, radiomics

## Abstract

**Background:**

This study aimed to develop and validate a nomogram integrating multimodal imaging and clinical features for the preoperative prediction of human epidermal growth factor receptor 2 (HER2) expression status in patients with invasive breast cancer.

**Methods:**

A total of 204 patients with pathologically confirmed breast cancer who underwent preoperative dynamic contrast-enhanced magnetic resonance imaging (DCE-MRI) and mammography (MG) were retrospectively enrolled. Patients were randomly divided into training and test sets in a 7:3 ratio. Pearson correlation coefficient (PCC) combined with recursive feature elimination (RFE) was used to select optimal radiomics features. Three binary classification tasks were constructed (Model_1: HER2-Positive vs. HER2-Zero; Model_2: HER2-Positive vs. HER2-Low; Model_3: HER2-Low vs. HER2-Zero), each containing three sub-models based on MG, DCE-MRI, and their combination. The optimal radiomics model for each task was selected and integrated with independent clinicopathological factors to establish a clinical-radiomics nomogram.

**Results:**

The combined radiomics model achieved the best performance for Model_1 and Model_2, with area under the curve (AUC) values of 0.742 (95% confidence interval [CI]: 0.624–0.860) and 0.823 (95% CI: 0.749–0.897) in the training set, and 0.718 (95% CI: 0.594–0.842) and 0.778 (95% CI: 0.696–0.861) in the validation set, respectively. Corresponding nomograms were subsequently constructed incorporating independent clinical predictors. For Model_3, the single DCE-MRI model demonstrated optimal performance (training AUC = 0.831, 95% CI: 0.747–0.916; validation AUC = 0.745, 95% CI: 0.640–0.850). However, no nomogram was developed due to the absence of significant clinicopathological features (p > 0.05).

**Conclusion:**

The multimodal radiomics-based nomogram, integrating imaging features and clinical factors, provides a promising non-invasive quantitative tool for preoperative evaluation of HER2 expression status in breast cancer, offering significant potential for clinical translation.

## 1 Introduction

Breast cancer is the most common malignancy among women worldwide, with incidence and mortality rates ranking first in female cancers, accounting for approximately 31% of all cancer cases in women (1, 2). Approximately 20–30% of breast cancer patients exhibit overexpression of the human epidermal growth factor receptor 2 (HER2) (3). As a key oncogenic driver, HER2 regulates multiple signaling pathways involved in cell growth, differentiation, proliferation, and apoptosis, making it a critical therapeutic target (4–6). Accurate assessment of HER2 expression status is therefore essential for prognosis and treatment decision-making.

Currently, HER2 status is primarily determined via immunohistochemistry (IHC) and fluorescence *in situ* hybridization (FISH). However, these invasive methods are costly, time-consuming (7, 8), and biopsy samples may not fully capture intratumoral heterogeneity (9, 10). Thus, there is an urgent need for non-invasive techniques capable of distinguishing the three HER2 expression states. Radiomics offers a promising approach by quantitatively extracting pathophysiological features and heterogeneity information from medical images (11, 12). Recent studies have demonstrated that qualitative features from breast magnetic resonance imaging (MRI), mammography, and ultrasound can differentiate HER2 expression status (13–16). Nevertheless, existing research is largely limited to single-modality analyses, and studies combining DCE-MRI with mammography radiomics features to distinguish HER2-zero, HER2-low, and HER2-positive expression remain scarce.

Therefore, this study aimed to integrate MG and DCE-MRI radiomics features to develop a joint prediction model for differentiating HER2 expression status, thereby providing a non-invasive diagnostic tool to support preoperative evaluation and personalized treatment planning.

## 2 Materials and methods

### 2.1 Study population

**Figure 1:**
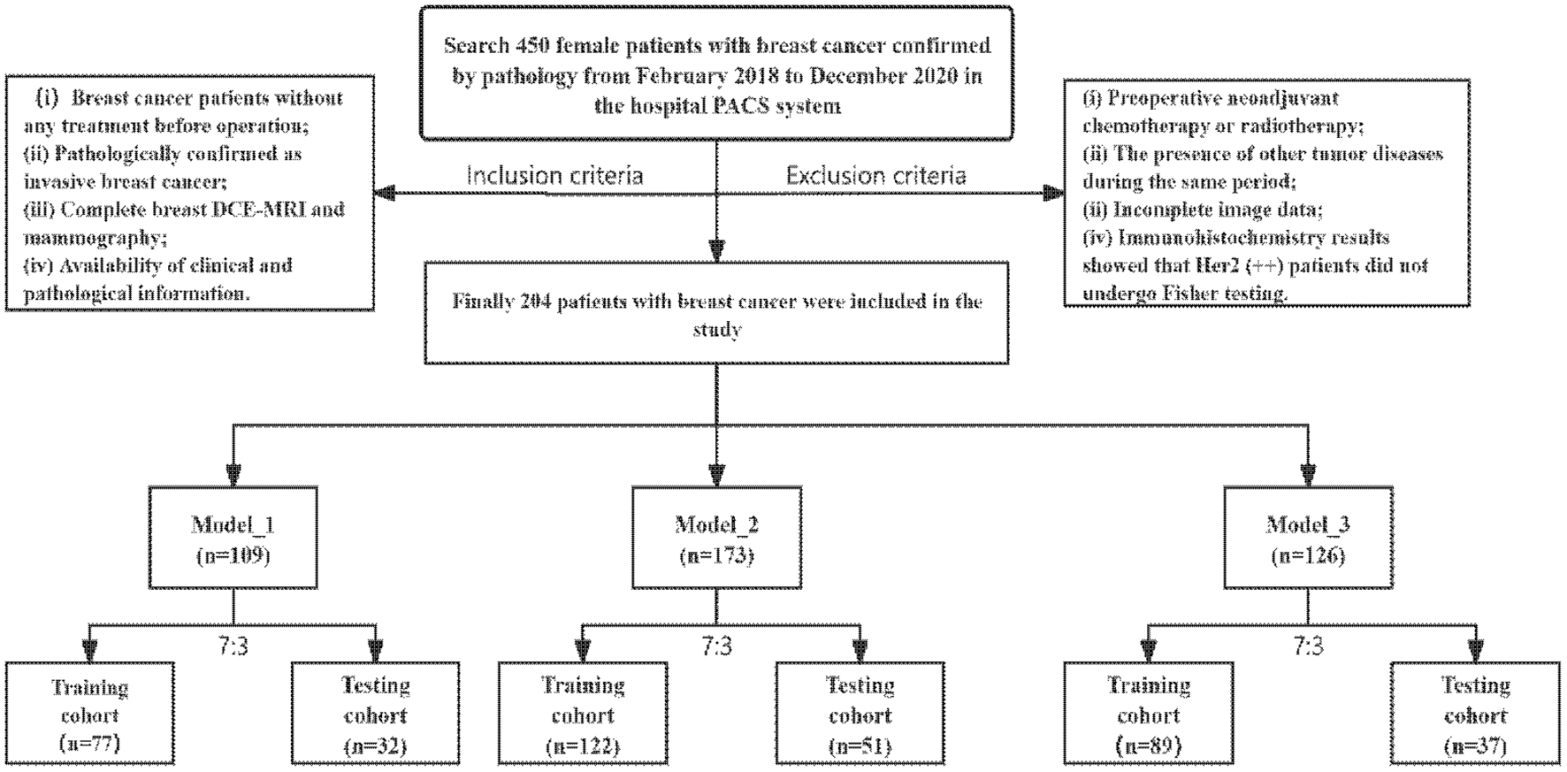
Flowchart of patient selection and enrollment.

This retrospective study was approved by the Ethics Committee of the Affiliated Hospital of Guizhou Medical University, and the requirement for informed consent was waived. A total of 450 female patients with pathologically confirmed breast cancer between February 2018 and December 2020 were retrospectively identified. Inclusion criteria were: (i) no preoperative treatment; (ii) pathologically confirmed invasive breast cancer; (iii) availability of complete preoperative breast DCE-MRI and MG data; and (iv) complete clinical and pathological records. Exclusion criteria were: (i) preoperative neoadjuvant radio-chemotherapy; (ii) presence of other malignancies; (iii) incomplete imaging data; (iv) non-invasive breast cancer; and (v) HER2 (2+) without FISH testing. A total of 204 patients (mean age 51.71 ± 10.17 years; range 30–87 years) were included and randomly divided into training and test sets in a 7:3 ratio. The patient recruitment flowchart is shown in **Figure 1**.

HER2 status was determined by IHC according to the 2018 ASCO/CAP guidelines (8). Patients were categorized into three groups: HER2-zero (IHC 0; n=31), HER2-low (IHC 1+ or IHC 2+ with FISH-negative; n=95), and HER2-positive (IHC 3+ or IHC 2+ with FISH-positive; n=78).

Clinicopathological features including age, menopausal status, history of miscarriage, Breast Imaging Reporting and Data System (BI-RADS) category, tumor location, estrogen receptor (ER), progesterone receptor (PR), Ki-67 index, and lymph node metastasis were extracted from medical records. Preoperative DCE-MRI and MG reports were also collected.

### 2.2 Image acquisition

Mammography was performed using the Selenia Dimensions system, with patients positioned for standard cranio-caudal (CC) and medio-lateral oblique (MLO) views. Local pressure magnification was applied when necessary, and images were acquired in automatic exposure mode.

Breast MRI was performed using Philips 3.0 T Achieva and Ingenia scanners (same manufacturer, same field strength, with standardized scanning sequences and post-processing to ensure data comparability). Patients were placed in a prone position with both breasts naturally dependent. Dynamic contrast-enhanced scans covered both breasts and axillae. Gadopentetate dimeglumine (469.01 mg/mL) was injected intravenously at a rate of 2.0 mL/s (0.1 mL/kg), followed by a 15 mL saline flush at the same rate. Sequence parameters for the axial dynamic contrast-enhanced sequence were: repetition time (TR)=4.1 ms, echo time (TE)=2.1 ms, flip angle (FA)=8°, slice thickness=1 mm, slice spacing=0 mm. Eight consecutive phases were acquired, each lasting 50 seconds.

### 2.3 Image segmentation

**Figure 2:**
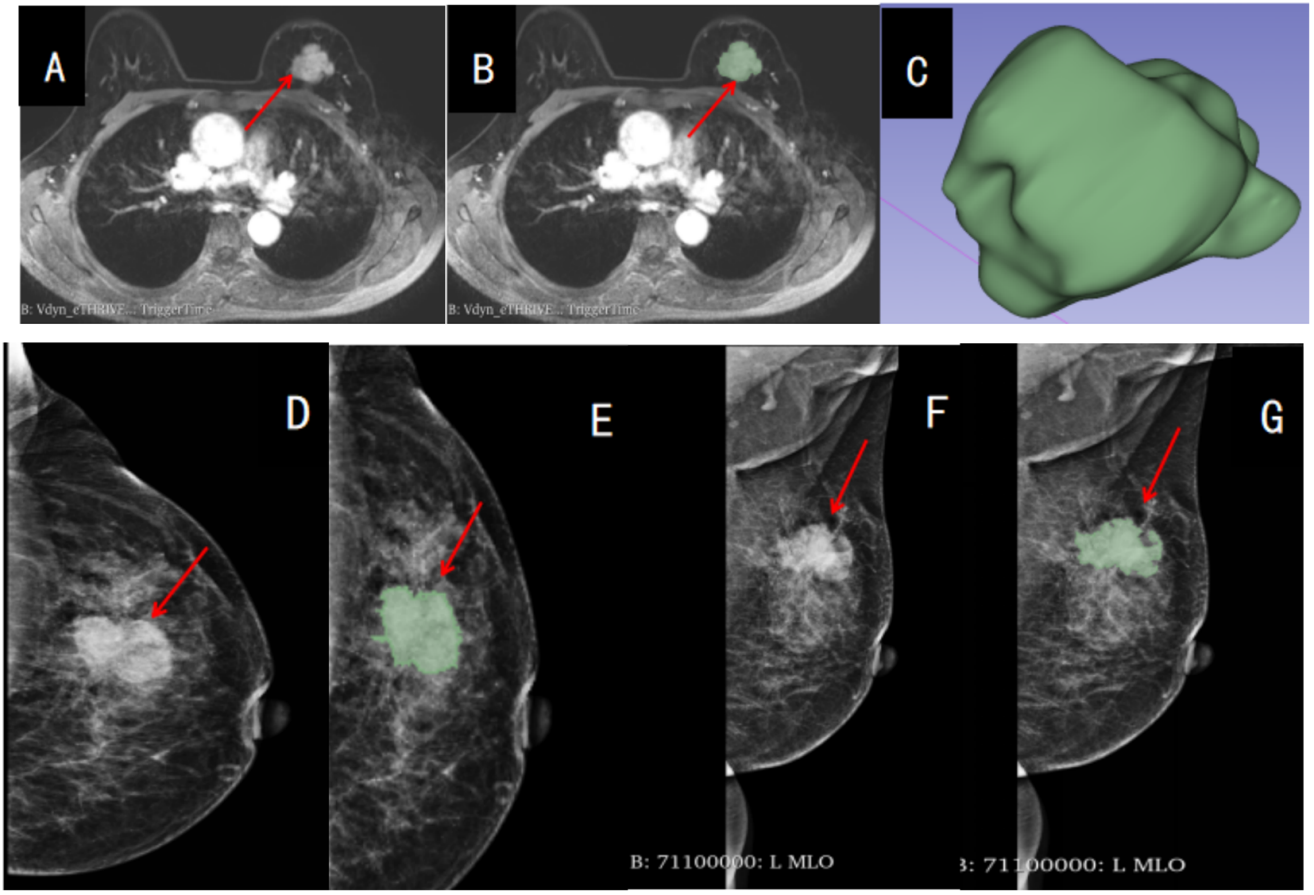
Representative images from a 52-year-old female with left breast invasive carcinoma (HER2-positive). **(A–C)** Contrast-enhanced T1-weighted MRI with sequential slice-by-slice delineation. **(A, B)** Axial images show a lobulated, markedly enhancing mass (red arrows). **(C)** Fused 3D ROI reconstruction. **(D, E)** CC view mammography: **(D)** Irregular lobulated mass in the upper central left breast (red arrow); **(E)** Corresponding ROI delineation. **(F, G)** MLO view: **(F)** Same mass as in panel **(D)** (red arrow); **(G)** ROI delineation.

Imaging data were retrieved from the hospital’s Picture Archiving and Communication System (PACS). Segmentation was independently performed by experienced radiologists (with over 5 years of experience) using 3D Slicer software. For mammography, lesion boundaries were manually delineated on CC or MLO views to obtain 2D regions of interest (ROIs). For DCE-MRI, ROIs were delineated layer by layer and fused to generate 3D volumes of interest (VOIs), encompassing all enhancing regions (**Figure 2**). In cases of multifocal/multicentric disease, the largest lesion was selected for segmentation. To evaluate interobserver agreement, this consistency was assessed by two independent radiologists prior to feature extraction. The intraclass correlation coefficients (ICCs) for DCE-MRI and mammography were 0.894 and 0.842, respectively, both exceeding the recommended threshold of 0.75, indicating good reproducibility of the extracted radiomics features.

### 2.4 Radiomics feature extraction and selection

Radiomics features were extracted using the Pyradiomics module in 3D Slicer and analyzed with Feature Explorer Pro (FAE, V0.6.3, Python 3.7.6). Extracted features included first-order statistics, shape-based features, and texture features. Redundant features were removed using Pearson correlation coefficient (PCC), and the feature matrix was standardized by subtracting the mean and dividing by the standard deviation. Recursive feature elimination (RFE) was then applied for feature selection. Logistic regression (LR) with ten-fold cross-validation on the training set was used to determine the optimal feature subset based on model performance.

### 2.5 Model construction

Given that HER2-zero cases accounted for only 15.2% (31/204) of the cohort, the synthetic minority oversampling technique (SMOTE) was applied to balance class distribution in the training set (17). Z-score normalization was used to standardize feature ranges. Three classification tasks were defined, each with three sub-models (MG-only, DCE-MRI-only, and combined modality) using logistic regression: Model_1 (HER2-Positive vs. HER2-Zero), Model_2 (HER2-Positive vs. HER2-Low), and Model_3 (HER2-Low vs. HER2-Zero).To construct the combined model, all features from both modalities were concatenated into a single feature matrix, followed by unified feature selection and model building using the same methodology as applied to the single-modality models.

For each task, the model with the highest AUC in ten-fold cross-validation was selected as the candidate model (18). Univariate analysis was performed on nine clinical variables (age, menopausal status, miscarriage history, BI-RADS category, tumor location, ER, PR, Ki-67, and lymph node metastasis). Variables with significant intergroup differences were entered into multivariate logistic regression to identify independent predictors. Finally, a clinical-radiomics nomogram was constructed by integrating independent clinical predictors with the selected radiomics features.

### 2.6 Statistical analysis

Categorical variables were presented as frequencies (percentages) and compared using the chi-square test or Fisher’s exact test. Continuous variables were compared using the independent samples t-test (for normally distributed data) or the Mann-Whitney U test (for non-normally distributed data). Model performance was evaluated using receiver operating characteristic (ROC) curves. Nomograms were visualized to support clinical decision-making, and calibration curves were plotted to assess model fit. A two-tailed p-value < 0.05 was considered statistically significant. All statistical analyses were performed using R software (version 3.4.3), FAE, and SPSS (version 27.0).

## 3 Results

### 3.1 Clinicopathological characteristics

The clinicopathological characteristics of the training and test sets for Models 1–3 are summarized in **Tables 1**–**3**, respectively.

**Table 1:** Clinicopathological features of breast cancer patients in different sets for model_1.

	Training cohort (n=77)			Testing cohort (n=32)		
Characteristic	HER2-positive(n=55)	HER2-zero(n=22)	p	HER2-positive(n=23)	HER2-zero(n=9)	P
Age	49.58±10.43	52.18±9.21	0.311	49.87±9.06	62.56±11.57	0.003
side			0.773			0.444
Left	28 (50.9%)	12 (54.5%)		12 (52.2%)	3 (33.3%)	
Right	27 (49.1%)	10 (45.5%)		11 (47.8%)	6 (66.7%)	
Menopausal status			0.612			1.000
Pre	29 (52.7%)	13 (59.1%)		14 (60.9%)	5 (55.6%)	
Post	26 (47.3%)	9 (40.9%)		9 (39.1%)	4 (44.4%)	
History of miscarriage			0.545			1.000
Yes	20 (36.4%)	10 (45.5%)		7 (30.4%)	3 (33.3%)	
No	33 (60%)	12 (54.5%)		16 (69.6%)	6 (66.7%)	
Missing	2 (3.6%)	0 (0.0%)				
ER status			0.030			0.433
Negative	30 (54.5%)	6 (27.3%)		13 (56.5%)	3 (33.3%)	
Positive	25 (45.5%)	16 (72.7%)		10 (43.5%)	6 (66.7%)	
PR status			0.003			0.444
Negative	38 (69.1%)	7 (31.8%)		12 (52.2%)	3 (33.3%)	
Positive	17 (30.9%)	15 (68.2%)		11 (47.8%)	6 (66.7%)	
Ki-67 index			<0.001			0.003
<20%	3 (5.5%)	10 (45.5%)		1 (4.3%)	5 (55.6%)	
≥20%	52 (94.5%)	12 (54.5%)		22 (95.7%)	4 (44.4%)	
BI-RADS			0.432			0.636
4a	1 (1.8%)	1 (4.5%)		0 (0.0%)	1 (11.1%)	
4b	1 (1.8%)	2 (9.1%)		1 (4.3%)	0 (0.0%)	
4c	14 (25.5%)	5 (22.7%)		4 (17.4%)	1 (11.1%)	
5	39 (70.9%)	14 (63.6%)		18 (78.3%)	7 (77.8%)	
Pathologically confirmed axillary lymph node metastasis			0.075			0.151
Yes	29 (52.7%)	8 (36.4%)		8 (34.8%)	1 (11.1%)	
No	25 (45.5%)	11 (50.0%)		11 (47.8%)	8 (88.9%)	
Missing	1 (1.8%)	3 (13.6%)		4 (17.4%)	0 (0.0%)	

Pre, Premenopausal; Post, Postmenopausal; ER, Estrogen receptor; PR, Progesterone receptor; BI-RADS, Breast Imaging Reporting and Data System.

**Table 2:** Clinicopathological characteristics of patients with breast cancer in each set for model_2.

	Training cohort (n=122)			Testing cohort (n=51)		
Characteristic	HER2-positive (n=55)	HER2-low (n=67)	p	HER2-positive (n=23)	HER2-low (n=28)	P
Age	50.31±9.05	51.85±10.18	0.510	48.13±12.03	53.18±8.94	0.103
side			0.635			0.473
Left	27 (49.1%)	30 (44.8%)		13 (56.5%)	13 (46.4%)	
Right	28 (50.9%)	37 (55.2%)		10 (43.5%)	15 (53.6%)	
Menopausal status			0.940			0.473
Pre	30 (54.5%)	37 (55.2%)		13 (56.5%)	13 (46.4%)	
Post	25 (45.5%)	30 (44.8%)		10 (43.5%)	15 (53.6%)	
History of miscarriage			0.283			0.462
Yes	22 (40.7%)	21 (31.3%)		5 (22.7%)	9 (32.1%)	
No	32 (59.3%)	46 (68.7%)		17 (77.3%)	19 (67.9%)	
Missing	0	0		1 (4.3%)	0 (0.0%)	
ER status			<0.001			0.010
Negative	31 (56.4%)	10 (14.9%)		12 (52.2%)	5 (17.9%)	
Positive	24 (43.6%)	57 (85.1%)		11 (47.8%)	23 (82.1%)	
PR status			<0.001			0.003
Negative	33 (60.0%)	17 (25.4%)		17 (73.9%)	9 (32.1%)	
Positive	22 (40.0%)	50 (74.6%)		6 (26.1%)	19 (67.9%)	
Ki-67 index			<0.001			0.059
<20%	3 (5.5%)	21 (31.3%)		1 (4.3%)	8 (28.6%)	
≥20%	52 (94.5%)	46 (68.7%)		22 (95.7%)	20 (71.4%)	
BI-RADS			0.821			0.130
4a	1 (1.8%)	3 (4.5%)		0 (0.0%)	2 (7.1%)	
4b	2 (3.6%)	3 (4.5%)		0 (0.0%)	4 (14.3%)	
4c	11 (20.0%)	11 (16.4%)		7 (30.4%)	6 (21.4%)	
5	41 (74.5%)	50 (74.6%)		16 (69.6%)	16 (57.1%)	
Pathologically confirmed axillary lymph node metastasis			0.670			0.653
Yes	26 (47.3%)	29 (43.3%)		11 (47.8%)	10 (35.7%)	
No	25 (45.5%)	35 (52.2%)		11 (47.8%)	17 (60.7%)	
Missing	4 (7.3%)	3 (4.5%)		1 (4.3%)	1 (3.6%)	

Pre, Premenopausal; Post, Postmenopausal; ER, Estrogen receptor; PR, Progesterone receptor; BI-RADS, Breast Imaging Reporting and Data System.

**Table 3:** Clinicopathologic characteristics of patients with breast cancer in each set for model_3.

	Training cohort (n=89)			Testing cohort (n=37)		
Characteristic	HER2-Low (n=67)	HER2-Zero (n=22)	p	HER2-Low (n=28)	HER2-Zero (n=9)	P
Age	52.76±10.33	57.27±11.20	0.085	51.00±8.43	50.11±8.51	0.785
side			0.956			0.714
Left	30 (44.8%)	10 (45.5%)		13 (46.4%)	5 (55.6%)	
Right	37 (55.2%)	12 (54.5%)		15 (53.6%)	4 (44.4%)	
Menopausal status			0.947			0.462
Pre	36 (53.7%)	12 (54.5%)		14 (50.0%)	6 (66.7%)	
Post	31 (46.3%)	10 (45.5%)		14 (50.0%)	3 (33.3%)	
History of miscarriage			0.930			0.057
Yes	22 (32.8%)	7 (31.8%)		8 (28.6%)	6 (66.7%)	
No	45 (67.2%)	15 (68.2%)		20 (71.4%)	3 (33.3%)	
Missing	0 (0.0%)	0 (0.0%)		0 (0.0%)	0 (0.0%)	
ER status			0.210			0.620
Negative	11 (16.4%)	7 (31.8%)		4 (14.3%)	2 (22.2%)	
Positive	56 (83.6%)	15 (68.2%)		24 (85.7%)	7 (77.8%)	
PR status			0.654			1.000
Negative	18 (26.9%)	7 (31.8%)		8 (28.6%)	3 (33.3%)	
Positive	49 (73.1%)	15 (68.2%)		20 (71.4%)	6 (66.7%)	
Ki-67 index			0.114			0.432
<20%	46 (68.7%)	11 (50.0%)		8 (28.6%)	4 (44.4%)	
≥20%	21 (31.3%)	11 (50.0%)		20 (71.4%)	5 (55.6%)	
BI-RADS			0.491			0.449
4a	4 (6.0%)	2 (9.1%)		1 (3.6%)	0 (0.0%)	
4b	6 (9.0%)	0 (0.0%)		1 (3.6%)	2 (22.2%)	
4c	12 (17.9%)	5 (22.7%)		5 (17.9%)	1 (11.1%)	
5	45 (67.2%)	15 (68.2%)		21 (75.0%)	6 (66.7%)	
Pathologically confirmed axillary lymph node metastasis			0.401			0.330
Yes	29 (43.3%)	8 (36.4%)		10 (35.7%)	1 (11.1%)	
No	36 (53.7%)	12 (54.5%)		16 (57.1%)	7 (77.8%)	
Missing	2 (3.0%)	2 (9.1%)		2 (7.1%)	1 (11.1%)	

Pre, Premenopausal; Post, Postmenopausal; ER, Estrogen receptor; PR, Progesterone receptor; BI-RADS, Breast imaging reporting and data system.

For Model_1, the training set included 55 (71.4%) HER2-positive and 22 (28.6%) HER2-zero cases; the test set included 23 (71.9%) HER2-positive and 9 (28.1%) HER2-zero cases. ER, PR, and Ki-67 index differed significantly between groups in the training set (p < 0.05; **Table 1**). Multivariate analysis identified PR and Ki-67 as independent predictors.

For Model_2, the training set comprised 55 (45.1%) HER2-positive and 67 (54.9%) HER2-low cases; the test set comprised 23 (45.1%) HER2-positive and 28 (54.9%) HER2-low cases. Significant differences in ER, PR, and Ki-67 were observed in the training set (p < 0.05; **Table 2**). Multivariate analysis identified ER and Ki-67 as independent predictors.

For Model_3, the training set included 67 (75.3%) HER2-low and 22 (24.7%) HER2-zero cases; the test set included 28 (75.7%) HER2-low and 9 (24.3%) HER2-zero cases. No significant differences were found for any clinicopathological variables (p > 0.05; **Table 3**). Consequently, no independent clinical predictors were identified for this task.

### 3.2 Model development and validation

**Figure 3:**
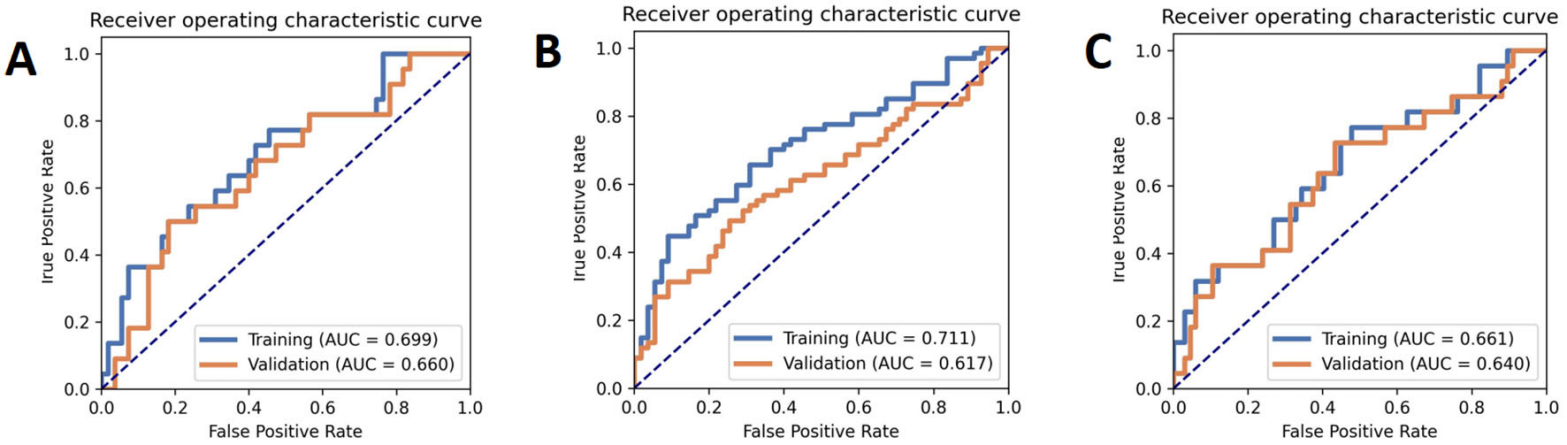
ROC curves of mammography (MG)-based radiomics models. **(A)** Model_1; **(B)** Model_2; **(C)** Model_3.

**Figure 4:**
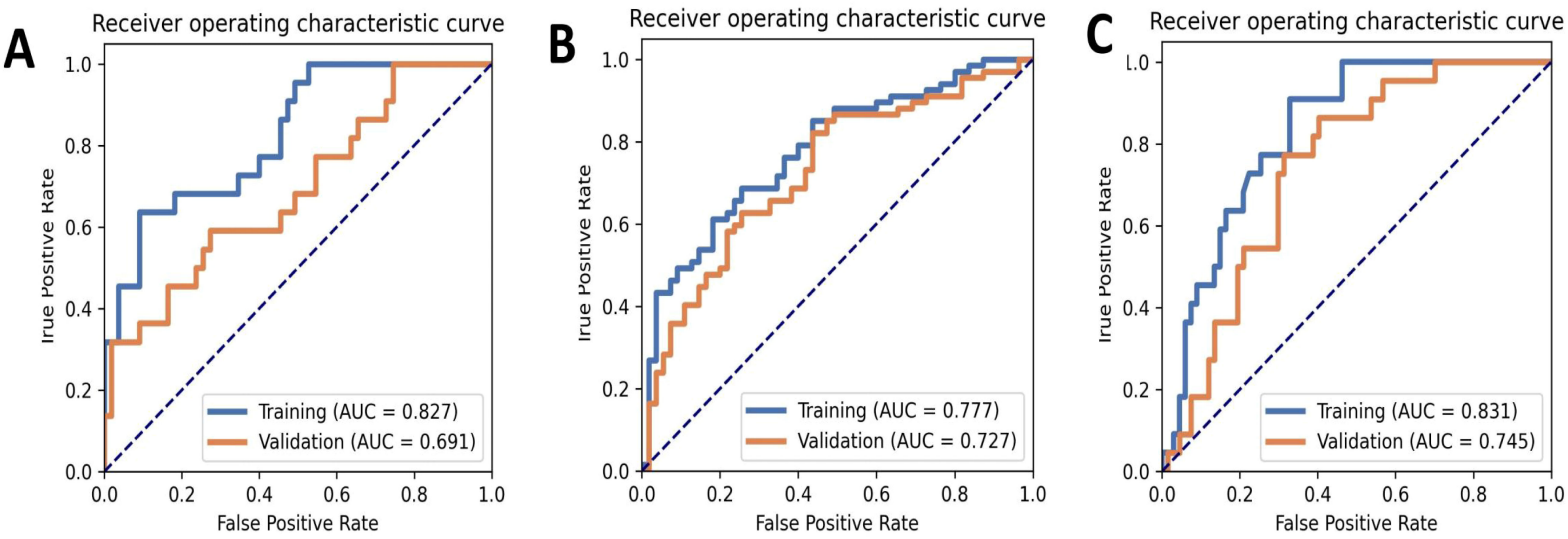
ROC curves of DCE-MRI-based radiomics models. **(A)** Model_1; **(B)** Model_2; **(C)** Model_3.

**Figure 5:**
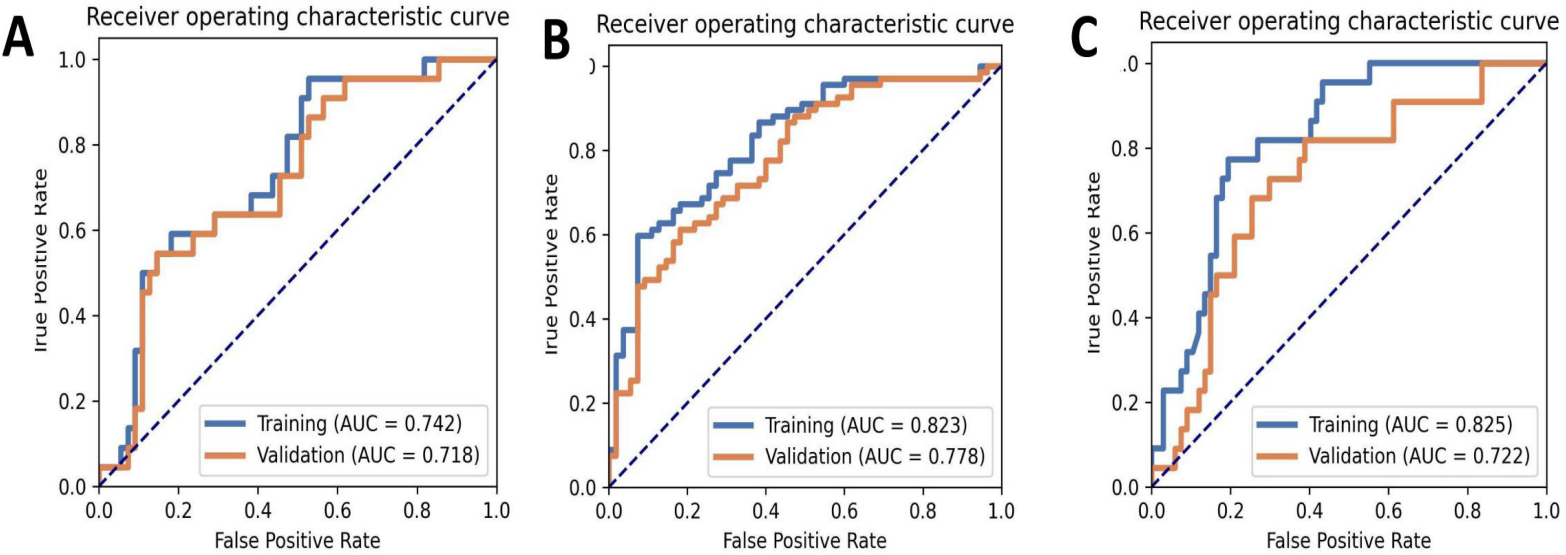
ROC curves of combined MG and DCE-MRI radiomics models. **(A)** Model_1; **(B)** Model_2; **(C)** Model_3.

Radiomics models based on MG, DCE-MRI, and their combination were constructed for each task. Predictive performance is illustrated in **Figures 3**–**5**.

#### 3.2.1 MG-based radiomics models

In the MG-based models, Model_1 (2 features) achieved an AUC of 0.660 (accuracy: 0.714) in cross-validation and 0.681 (accuracy: 0.656) in the test set (**Figure 3A**; **Table 4**). Model_2 (8 features) achieved a cross-validation AUC of 0.617 (accuracy: 0.566) and a test AUC of 0.702 (accuracy: 0.667) (**Figure 3B**; **Table 4**). Model_3 (1 feature) achieved a cross-validation AUC of 0.640 (accuracy: 0.764) and a test AUC of 0.681 (accuracy: 0.568) (**Figure 3C**; **Table 4**).

**Table 4:** Summary of predictive performance based on MG radiomics model.

	Training cohort					Validation cohort				
	AUC	95% CI	Accuracy	Sensitivity	Specificity	AUC	95% CI	Accuracy	Sensitivity	Specificity
Model_1	0.699	0.5672-0.8311	0.727	0.500	0.818	0.660	0.5231-0.7959	0.714	0.091	0.964
Model_2	0.711	0.6197-0.8023	0.664	0.642	0.691	0.617	0.5174-0.7168	0.566	0.657	0.455
Model_3	0.661	0.5240-0.7990	0.596	0.727	0.552	0.640	0.4989-0.7806	0.764	0.273	0.925

AUC Area Under the ROC Curve, 95% CI 95% confidence interval.

#### 3.2.2 DCE-MRI-based radiomics models

For DCE-MRI models, Model_1 (10 features) achieved a cross-validation AUC of 0.691 (accuracy: 0.753) and a test AUC of 0.686 (accuracy: 0.719) (**Figure 4A**; **Table 5**). Model_2 (5 features) achieved a cross-validation AUC of 0.727 (accuracy: 0.672) and a test AUC of 0.674 (accuracy: 0.588) (**Figure 4B**; **Table 5**). Model_3 (9 features) achieved a cross-validation AUC of 0.745 (accuracy: 0.697) and a test AUC of 0.730 (accuracy: 0.622) (**Figure 4C**; **Table 5**).

**Table 5:** Summary of radiomics feature prediction performance based on DCE-MRI model.

	Training cohort					Validation cohort				
	AUC	95% CI	Accuracy	Sensitivity	Specificity	AUC	95% CI	Accuracy	Sensitivity	Specificity
Model_1	0.827	0.7280-0.9265	0.831	0.636	0.909	0.691	0.5564-0.8254	0.753	0.364	0.909
Model_2	0.777	0.6955-0.8594	0.705	0.612	0.818	0.727	0.6364-0.8170	0.672	0.612	0.745
Model_3	0.831	0.7465-0.9164	0.730	0.909	0.672	0.745	0.6396-0.8503	0.697	0.546	0.746

AUC Area Under the ROC Curve, 95% CI 95% confidence interval.

#### 3.2.3 Combined MG and DCE-MRI radiomics models

For combined models, Model_1 (2 features) achieved a cross-validation AUC of 0.718 (accuracy: 0.688) and a test AUC of 0.609 (accuracy: 0.750) (**Figure 5A**; **Table 6**). Model_2 (9 features) achieved a cross-validation AUC of 0.778 (accuracy: 0.697) and a test AUC of 0.629 (accuracy: 0.588) (**Figure 5B**; **Table 6**). Model_3 (8 features) achieved a cross-validation AUC of 0.722 (accuracy: 0.753) and a test AUC of 0.635 (accuracy: 0.540) (**Figure 5C**; **Table 6**).

**Table 6:** Summary of radiomics feature prediction performance based on mammography combined with DCE-MRI model.

	Training cohort					Validation cohort				
	AUC	95% CI	Accuracy	Sensitivity	Specificity	AUC	95% CI	Accuracy	Sensitivity	Specificity
Model_1	0.742	0.6241-0.8602	0.766	0.500	0.873	0.718	0.5943-0.8421	0.688	0.091	0.927
Model_2	0.823	0.7486-0.8970	0.738	0.582	0.927	0.778	0.6955-0.8605	0.697	0.597	0.818
Model_3	0.825	0.7360-0.9133	0.753	0.818	0.731	0.722	0.6006-0.8431	0.753	0.500	0.836

AUC Area Under the ROC Curve, 95% CI 95% confidence interval.

A summary of radiomics model performance is provided in **Tables 4**-**6**. Based on validation set AUCs, the combined model performed best for Model_1 and Model_2. Specifically, Model_1 combined model yielded an AUC of 0.718 (95% CI: 0.594–0.842), accuracy of 0.688, sensitivity of 0.091, and specificity of 0.927; Model_2 combined model yielded an AUC of 0.778 (95% CI: 0.696–0.861), accuracy of 0.697, sensitivity of 0.597, and specificity of 0.818 (**Figures 5A, B**). These were therefore selected as candidate models.

## 4 Clinical application

**Figure 6:**
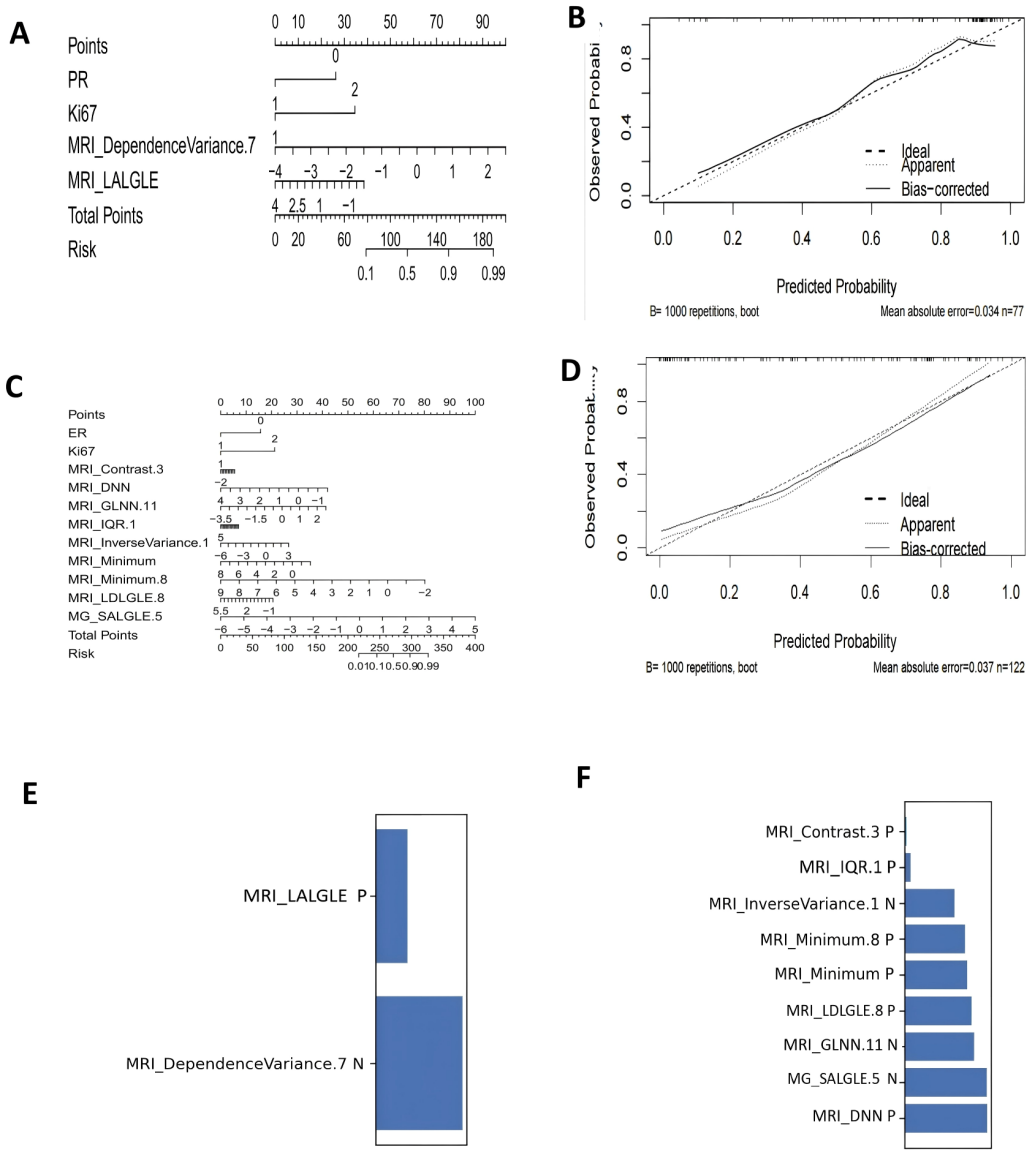
**(A)** Clinical-radiomics nomogram for Model_1 (incorporating 2 MRI radiomics features and 2 clinical features). **(B)** Calibration curve for Model_1 nomogram. **(C)** Clinical-radiomics nomogram for Model_2 (incorporating 8 MRI radiomics features, 1 mammography feature, and 2 clinical features). **(D)** Calibration curve for Model_2 nomogram. In calibration curves **(B, D)**, the x-axis represents predicted probability, the y-axis represents observed probability. The ideal line indicates perfect prediction; the apparent line represents the unadjusted fit; the bias-corrected line represents the fit after 1,000 bootstrap iterations. **(E, F)** Coefficient weight plots for Model_1 and Model_2 radiomics features. Feature names followed by “P” indicate positive correlation with HER2 status; “N” indicates negative correlation. MRI_LALGLE: MRI_SmallAreaLowGrayLevelEmphasis; MRI_DNN: MRI_DependenceNonUniformityNormalized; MRI_GLNN.11: MRI_GrayLevelNonUniformityNormalized.11; MRI_IQR.1: MRI_InterquartileRange.1; MRI_LDLGLE.8: MRI_SmallDependenceLowGrayLevelEmphasis.8; M_SALGLE.5: M_SmallAreaLowGrayLevelEmphasis.5.

For Model_1, a clinical-radiomics nomogram was constructed using two radiomics features and two clinical features (PR and Ki-67) (**Figure 6A**). For Model_2, the nomogram incorporated nine radiomics features and two clinical features (ER and Ki-67) (**Figure 6C**). No nomogram was developed for Model_3 due to the absence of significant clinical predictors.

## 5 Discussion

Traditionally, HER2 expression has been categorized as positive or negative based on IHC scoring (19). However, the HER2-low subtype has recently been recognized, accounting for approximately 45–60% of HER2-negative tumors (20–22). With the identification of this subtype and the demonstrated efficacy of antibody-drug conjugates in HER2-low patients (23), the clinical demand for non-invasive preoperative differentiation of HER2 expression states has significantly increased. Radiomics offers a technical solution by quantifying tumor heterogeneity. In this single-center retrospective study of 204 invasive breast cancer patients, predictive models for three binary classification tasks (HER2-Positive vs. HER2-Zero; HER2-Positive vs. HER2-Low; HER2-Low vs. HER2-Zero) were developed based on DCE-MRI, MG, and clinical features. Combined modality models outperformed single−modality models for Model_1 and Model_2, yielding validation AUCs of 0.718 and 0.778, respectively. Corresponding nomograms integrating clinical predictors were constructed. For Model_3, the single DCE-MRI model was optimal (validation AUC = 0.745), but no combined nomogram was developed due to the lack of significant clinical predictors. These findings suggest that multimodal radiomics combined with clinical features holds promise for individualized preoperative prediction of HER2 expression status, particularly in patients with equivocal IHC results or limited access to FISH testing.

Previous studies have developed radiomics models to distinguish HER2-zero, HER2-low, and HER2-positive breast cancer (14, 16, 24, 25). While these studies confirmed the potential of radiomics, most were limited to single-modality analysis. This study innovatively integrates dual−modality imaging (MG and DCE−MRI) to capture multidimensional tumor information, potentially overcoming the limitations of single−modality approaches and providing a more comprehensive characterization of tumor heterogeneity. Furthermore, prior research has largely focused on binary classifications (positive/negative or low/zero), with relatively few studies addressing the distinction between low and zero expression in the context of HER2 positivity. For Model_1 and Model_2, the combined model outperformed previously reported single-modality models. For example, Chen et al. (14) developed a multiparametric MRI model with an AUC of 0.624, whereas the combined model achieved validation AUCs of 0.718 and 0.778, respectively. For the low vs. zero expression task, Chen H et al. (25) quantified intratumoral heterogeneity and reported AUCs of 0.94, 0.93, and 0.84 in training, validation, and test sets. In comparison, the combined model for this task yielded lower AUCs (training: 0.825, validation: 0.722, test: 0.635), which may be attributable to the relatively small sample size, class imbalance, and limited model stability. It was also observed that the diagnostic performance for Model_1 was inferior to that for Model_2 and Model_3, suggesting that biological heterogeneity between HER2−positive and HER2−zero tumors may be relatively low, warranting further investigation. Consistent with prior research (26), significant associations between HER2 status and ER, PR, and Ki−67 expression were found, supporting the biological relevance of these markers in HER2−driven breast cancer.

Despite its promising findings, this study has several limitations. First, as a retrospective single-center study with a standardized imaging protocol, the model may have limited generalizability to other institutions or scanner platforms (27, 28). Multicenter external validation is necessary to assess robustness and transportability. Second, although independent test sets were used, model performance—particularly for Model_3—requires further improvement through larger-scale internal and external validation. Third, the modest sample size and imbalanced class distribution increase the risk of overfitting and prediction bias. Future studies should include larger, more balanced cohorts and incorporate multicenter data to enhance model reproducibility and clinical applicability. Fourth, due to the retrospective design, key clinical covariates (family history, parity, and breastfeeding history) were unavailable, precluding their inclusion in multivariable analysis. This may partly explain the absence of independent predictors in Model_3 (HER2-Low vs. HER2-Zero). Prospective studies with larger cohorts are needed to validate their added value to this nomogram. Finally, mammography lesions were segmented using 2D ROIs, which may be susceptible to partial volume effects. Future work should explore 3D segmentation techniques to more comprehensively capture tumor characteristics.

In conclusion, these findings indicate that radiomics features derived from mammography combined with DCE-MRI, integrated with clinical factors, offer valuable clinical guidance for predicting HER2 expression status in breast cancer and provide a promising non-invasive tool for identifying patients who may benefit from HER2-targeted therapies.

## Data Availability

The original contributions presented in the study are included in the article/supplementary material, further inquiries can be directed to the corresponding author/s.
